# Supplementary material for: Spectral signature and behavioral consequence of spontaneous shifts of pupil-linked arousal in human
Source: eLife. 2021 Aug 31;10:e68265. doi: 10.7554/eLife.68265 (PMC8486382; doi:10.7554/eLife.68265)
Supplement: Supplementary file 1. [file elife-68265-supp1.docx]

**Supplementary table 1**

|  | **experiment** | **Frequency**  **band** | **RSN** | $\chi_{Q}$ | **Q_pval** | **Q_std** | $\chi_{L}$ | **L_pval** | **L_std** | **BIC**  **(eq. 4)** | **BIC**  **(eq. 3)** | **L**  **Marg R^2^** | **Q**  **Marg R^2^** | **L**  **Cond R^2^** | **Q**  **Cond R^2^** |
| --- | --- | --- | --- | --- | --- | --- | --- | --- | --- | --- | --- | --- | --- | --- | --- |
| **1** | task_prestim | delta | Vis | nan | nan | nan | -0.017 | 0.079 | 0.010 | 5443.9 | 5448.6 | 0.003 | nan | 0.020 | nan |
| **2** | task_prestim | delta | SM | nan | nan | nan | -0.031 | 0.000 | 0.008 | 4549.7 | 4566.3 | 0.010 | nan | 0.023 | nan |
| **3** | task_prestim | delta | DAN | nan | nan | nan | -0.017 | 0.060 | 0.008 | 4957.0 | 4976.5 | 0.003 | nan | 0.016 | nan |
| **4** | task_prestim | delta | VAN | nan | nan | nan | -0.033 | 0.000 | 0.008 | 3770.8 | 3776.6 | 0.012 | nan | 0.027 | nan |
| **5** | task_prestim | delta | Lim | 0.018 | 0.000 | 0.005 | -0.022 | 0.079 | 0.012 | 10696.7 | 10677.7 | nan | 0.007 | nan | 0.024 |
| **6** | task_prestim | delta | FPN | nan | nan | nan | -0.028 | 0.004 | 0.009 | 4574.4 | 4583.7 | 0.007 | nan | 0.023 | nan |
| **7** | task_prestim | delta | DMN | nan | nan | nan | -0.026 | 0.008 | 0.009 | 3902.5 | 3907.2 | 0.007 | nan | 0.025 | nan |
| **8** | task_prestim | theta | Vis | -0.009 | 0.027 | 0.004 | -0.003 | 0.942 | 0.011 | 7123.2 | 7121.1 | nan | 0.002 | nan | 0.024 |
| **9** | task_prestim | theta | SM | nan | nan | nan | 0.006 | 0.817 | 0.007 | 5515.1 | 5515.3 | 0.000 | nan | 0.008 | nan |
| **10** | task_prestim | theta | DAN | -0.011 | 0.005 | 0.004 | 0.015 | 0.225 | 0.007 | 6692.9 | 6686.4 | nan | 0.004 | nan | 0.009 |
| **11** | task_prestim | theta | VAN | nan | nan | nan | 0.001 | 0.942 | 0.006 | 4240.4 | 4247.8 | 0.000 | nan | 0.005 | nan |
| **12** | task_prestim | theta | Lim | nan | nan | nan | -0.009 | 0.817 | 0.008 | 9345.6 | 9368.3 | 0.001 | nan | 0.006 | nan |
| **13** | task_prestim | theta | FPN | nan | nan | nan | 0.004 | 0.817 | 0.006 | 4898.5 | 4910.4 | 0.000 | nan | 0.005 | nan |
| **14** | task_prestim | theta | DMN | nan | nan | nan | 0.000 | 0.942 | 0.006 | 4496.6 | 4499.7 | 0.000 | nan | 0.007 | nan |
| **15** | task_prestim | alpha | Vis | -0.017 | 0.000 | 0.004 | 0.032 | 0.004 | 0.011 | 9438.4 | 9425.2 | nan | 0.010 | nan | 0.021 |
| **16** | task_prestim | alpha | SM | -0.013 | 0.000 | 0.003 | 0.024 | 0.005 | 0.009 | 5382.1 | 5362.4 | nan | 0.010 | nan | 0.033 |
| **17** | task_prestim | alpha | DAN | -0.017 | 0.000 | 0.003 | 0.035 | 0.000 | 0.008 | 6446.7 | 6413.9 | nan | 0.017 | nan | 0.035 |
| **18** | task_prestim | alpha | VAN | -0.011 | 0.000 | 0.003 | 0.025 | 0.003 | 0.008 | 3654.3 | 3640.1 | nan | 0.010 | nan | 0.037 |
| **19** | task_prestim | alpha | Lim | nan | nan | nan | 0.018 | 0.032 | 0.008 | 7693.8 | 7711.7 | 0.002 | nan | 0.010 | nan |
| **20** | task_prestim | alpha | FPN | -0.011 | 0.000 | 0.003 | 0.028 | 0.001 | 0.008 | 4093.3 | 4081.3 | nan | 0.012 | nan | 0.036 |
| **21** | task_prestim | alpha | DMN | -0.013 | 0.000 | 0.003 | 0.025 | 0.002 | 0.007 | 3922.6 | 3899.8 | nan | 0.012 | nan | 0.033 |
| **22** | task_prestim | beta | Vis | nan | nan | nan | 0.034 | 0.000 | 0.007 | 1609.8 | 1615.8 | 0.016 | nan | 0.029 | nan |
| **23** | task_prestim | beta | SM | -0.007 | 0.003 | 0.002 | 0.043 | 0.000 | 0.007 | 609.0 | 607.2 | nan | 0.028 | nan | 0.043 |
| **24** | task_prestim | beta | DAN | nan | nan | nan | 0.045 | 0.000 | 0.006 | 452.4 | 454.8 | 0.032 | nan | 0.045 | nan |
| **25** | task_prestim | beta | VAN | nan | nan | nan | 0.035 | 0.000 | 0.006 | -1718.6 | -1715.1 | 0.024 | nan | 0.039 | nan |
| **26** | task_prestim | beta | Lim | nan | nan | nan | 0.022 | 0.000 | 0.006 | 3826.2 | 3847.4 | 0.005 | nan | 0.011 | nan |
| **27** | task_prestim | beta | FPN | nan | nan | nan | 0.031 | 0.000 | 0.005 | -1344.9 | -1333.1 | 0.019 | nan | 0.031 | nan |
| **28** | task_prestim | beta | DMN | nan | nan | nan | 0.031 | 0.000 | 0.005 | -2773.6 | -2765.2 | 0.022 | nan | 0.034 | nan |
| **29** | task_prestim | gamma | Vis | 0.009 | 0.000 | 0.002 | 0.017 | 0.000 | 0.004 | -3866.4 | -3900.1 | nan | 0.013 | nan | 0.029 |
| **30** | task_prestim | gamma | SM | 0.008 | 0.000 | 0.002 | 0.028 | 0.000 | 0.005 | -630.5 | -633.7 | nan | 0.017 | nan | 0.031 |
| **31** | task_prestim | gamma | DAN | 0.008 | 0.000 | 0.002 | 0.023 | 0.000 | 0.004 | -3558.8 | -3569.2 | nan | 0.017 | nan | 0.023 |
| **32** | task_prestim | gamma | VAN | 0.008 | 0.000 | 0.002 | 0.027 | 0.000 | 0.005 | -671.7 | -678.9 | nan | 0.017 | nan | 0.031 |
| **33** | task_prestim | gamma | Lim | 0.009 | 0.002 | 0.003 | 0.030 | 0.000 | 0.006 | 3622.9 | 3614.2 | nan | 0.012 | nan | 0.022 |
| **34** | task_prestim | gamma | FPN | 0.007 | 0.000 | 0.002 | 0.021 | 0.000 | 0.004 | -2554.2 | -2558.0 | nan | 0.014 | nan | 0.025 |
| **35** | task_prestim | gamma | DMN | 0.008 | 0.000 | 0.002 | 0.022 | 0.000 | 0.004 | -2664.8 | -2680.7 | nan | 0.015 | nan | 0.029 |
| **36** | rest | delta | Vis | nan | nan | nan | -0.004 | 0.616 | 0.007 | 1734.0 | 1707.5 | 0.000 | nan | 0.010 | nan |
| **37** | rest | delta | SM | nan | nan | nan | -0.009 | 0.545 | 0.007 | 187.9 | 193.9 | 0.001 | nan | 0.019 | nan |
| **38** | rest | delta | DAN | nan | nan | nan | 0.002 | 0.804 | 0.008 | 882.5 | 885.4 | 0.000 | nan | 0.017 | nan |
| **39** | rest | delta | VAN | 0.008 | 0.003 | 0.003 | -0.011 | 0.201 | 0.006 | -191.1 | -191.2 | nan | 0.005 | nan | 0.026 |
| **40** | rest | delta | Lim | 0.018 | 0.000 | 0.003 | -0.014 | 0.201 | 0.007 | 2471.6 | 2429.5 | nan | 0.010 | nan | 0.026 |
| **41** | rest | delta | FPN | nan | nan | nan | -0.004 | 0.616 | 0.006 | 126.1 | 126.9 | 0.000 | nan | 0.012 | nan |
| **42** | rest | delta | DMN | 0.008 | 0.012 | 0.003 | -0.006 | 0.566 | 0.006 | 199.2 | 189.6 | nan | 0.003 | nan | 0.029 |
| **43** | rest | theta | Vis | nan | nan | nan | 0.008 | 0.529 | 0.010 | 3173.0 | 3155.0 | 0.001 | nan | 0.024 | nan |
| **44** | rest | theta | SM | nan | nan | nan | 0.009 | 0.529 | 0.010 | 1684.6 | 1688.1 | 0.001 | nan | 0.030 | nan |
| **45** | rest | theta | DAN | -0.010 | 0.008 | 0.004 | 0.021 | 0.506 | 0.012 | 3110.9 | 3097.9 | nan | 0.006 | nan | 0.035 |
| **46** | rest | theta | VAN | nan | nan | nan | 0.008 | 0.529 | 0.009 | 1132.0 | 1140.0 | 0.001 | nan | 0.022 | nan |
| **47** | rest | theta | Lim | nan | nan | nan | -0.002 | 0.702 | 0.006 | 1466.5 | 1475.8 | 0.000 | nan | 0.009 | nan |
| **48** | rest | theta | FPN | nan | nan | nan | 0.011 | 0.529 | 0.009 | 1393.0 | 1397.6 | 0.002 | nan | 0.024 | nan |
| **49** | rest | theta | DMN | nan | nan | nan | 0.010 | 0.529 | 0.009 | 1546.9 | 1546.2 | 0.001 | nan | 0.024 | nan |
| **50** | rest | alpha | Vis | -0.016 | 0.000 | 0.004 | 0.046 | 0.005 | 0.014 | 5260.7 | 5233.2 | nan | 0.018 | nan | 0.051 |
| **51** | rest | alpha | SM | -0.010 | 0.000 | 0.002 | 0.018 | 0.068 | 0.009 | 1492.8 | 1486.4 | nan | 0.007 | nan | 0.027 |
| **52** | rest | alpha | DAN | -0.016 | 0.000 | 0.003 | 0.034 | 0.005 | 0.011 | 2915.7 | 2875.0 | nan | 0.017 | nan | 0.040 |
| **53** | rest | alpha | VAN | -0.009 | 0.000 | 0.002 | 0.018 | 0.023 | 0.007 | 466.5 | 461.4 | nan | 0.008 | nan | 0.030 |
| **54** | rest | alpha | Lim | nan | nan | nan | 0.007 | 0.218 | 0.006 | 792.3 | 807.7 | 0.001 | nan | 0.009 | nan |
| **55** | rest | alpha | FPN | -0.010 | 0.000 | 0.003 | 0.023 | 0.007 | 0.008 | 647.2 | 634.5 | nan | 0.011 | nan | 0.037 |
| **56** | rest | alpha | DMN | -0.011 | 0.000 | 0.003 | 0.026 | 0.005 | 0.008 | 1129.9 | 1111.7 | nan | 0.013 | nan | 0.036 |
| **57** | rest | beta | Vis | nan | nan | nan | 0.027 | 0.001 | 0.008 | -1019.2 | -1023.3 | 0.014 | nan | 0.041 | nan |
| **58** | rest | beta | SM | nan | nan | nan | 0.022 | 0.003 | 0.007 | -2512.8 | -2502.1 | 0.012 | nan | 0.040 | nan |
| **59** | rest | beta | DAN | -0.009 | 0.000 | 0.002 | 0.035 | 0.000 | 0.008 | -2234.4 | -2242.1 | nan | 0.028 | nan | 0.066 |
| **60** | rest | beta | VAN | nan | nan | nan | 0.021 | 0.001 | 0.006 | -3771.2 | -3759.2 | 0.013 | nan | 0.036 | nan |
| **61** | rest | beta | Lim | nan | nan | nan | 0.014 | 0.001 | 0.004 | -4633.0 | -4624.3 | 0.007 | nan | 0.019 | nan |
| **62** | rest | beta | FPN | nan | nan | nan | 0.023 | 0.001 | 0.006 | -3688.6 | -3676.3 | 0.015 | nan | 0.037 | nan |
| **63** | rest | beta | DMN | nan | nan | nan | 0.025 | 0.000 | 0.006 | -4050.2 | -4039.5 | 0.018 | nan | 0.042 | nan |
| **64** | rest | gamma | Vis | 0.009 | 0.000 | 0.001 | 0.009 | 0.005 | 0.003 | -9567.4 | -9643.2 | nan | 0.027 | nan | 0.069 |
| **65** | rest | gamma | SM | 0.008 | 0.000 | 0.002 | 0.018 | 0.000 | 0.004 | -5336.1 | -5368.1 | nan | 0.025 | nan | 0.062 |
| **66** | rest | gamma | DAN | 0.007 | 0.000 | 0.001 | 0.014 | 0.000 | 0.003 | -8334.6 | -8365.0 | nan | 0.025 | nan | 0.059 |
| **67** | rest | gamma | VAN | 0.010 | 0.000 | 0.002 | 0.017 | 0.000 | 0.004 | -4738.5 | -4779.5 | nan | 0.024 | nan | 0.061 |
| **68** | rest | gamma | Lim | 0.014 | 0.000 | 0.003 | 0.022 | 0.001 | 0.006 | -1718.9 | -1786.1 | nan | 0.028 | nan | 0.074 |
| **69** | rest | gamma | FPN | 0.009 | 0.000 | 0.002 | 0.014 | 0.000 | 0.004 | -7001.4 | -7047.8 | nan | 0.025 | nan | 0.059 |
| **70** | rest | gamma | DMN | 0.010 | 0.000 | 0.002 | 0.014 | 0.000 | 0.004 | -6319.3 | -6383.1 | nan | 0.027 | nan | 0.068 |
| Supplementary table 1. Statistics for Figure 3 and Figure 3-figure supplement 1 reported for the model with the lowest BIC for each frequency band and RSN in task baseline and at rest. BIC is reported for both model types. p values are uncorrected, see the main results for significance after FDR correction across RSNs. Marg R^2^: marginal R^2^; Cond R^2^: conditional R^2^. | | | | | | | | | | | | | | | |

**Supplementary table 2**

|  | **BHV** | **fband** | **roi** | **Coef** | **pval** | **std** | **bic** | **Marginal R^2^** | **Conditional R^2^** |
| --- | --- | --- | --- | --- | --- | --- | --- | --- | --- |
| **1** | HR | delta | Vis | -0.029 | 0.001 | 0.009 | -180.203 | 0.028 | 0.857 |
| **2** | HR | delta | SM | 0.009 | 0.372 | 0.010 | -170.363 | 0.001 | 0.708 |
| **3** | HR | delta | DAN | 0.048 | 0.039 | 0.023 | 46.138 | 0.011 | 0.866 |
| **4** | HR | delta | VAN | -0.075 | 0.035 | 0.036 | 187.090 | 0.018 | 0.518 |
| **5** | HR | delta | Lim | -0.022 | 0.001 | 0.007 | -233.880 | 0.036 | 0.734 |
| **6** | HR | delta | FPN | 0.008 | 0.204 | 0.006 | -218.357 | 0.002 | 0.868 |
| **7** | HR | delta | DMN | -0.031 | 0.001 | 0.009 | -192.419 | 0.029 | 0.890 |
| **8** | HR | theta | Vis | -0.002 | 0.871 | 0.009 | -124.045 | 0.000 | 0.613 |
| **9** | HR | theta | SM | 0.043 | 0.099 | 0.026 | 91.535 | 0.008 | 0.790 |
| **10** | HR | theta | DAN | -0.098 | 0.014 | 0.040 | 194.344 | 0.028 | 0.550 |
| **11** | HR | theta | VAN | -0.020 | 0.009 | 0.008 | -243.160 | 0.027 | 0.799 |
| **12** | HR | theta | Lim | 0.014 | 0.035 | 0.007 | -192.855 | 0.007 | 0.833 |
| **13** | HR | theta | FPN | -0.024 | 0.009 | 0.009 | -204.148 | 0.018 | 0.898 |
| **14** | HR | theta | DMN | 0.008 | 0.407 | 0.010 | -142.807 | 0.002 | 0.755 |
| **15** | HR | alpha | Vis | 0.018 | 0.457 | 0.025 | 70.108 | 0.001 | 0.850 |
| **16** | HR | alpha | SM | -0.100 | 0.009 | 0.038 | 187.159 | 0.031 | 0.570 |
| **17** | HR | alpha | DAN | -0.013 | 0.022 | 0.006 | -238.881 | 0.012 | 0.727 |
| **18** | HR | alpha | VAN | 0.013 | 0.143 | 0.009 | -235.891 | 0.005 | 0.923 |
| **19** | HR | alpha | Lim | -0.024 | 0.005 | 0.008 | -194.549 | 0.018 | 0.885 |
| **20** | HR | alpha | FPN | -0.007 | 0.483 | 0.011 | -132.347 | 0.002 | 0.649 |
| **21** | HR | alpha | DMN | 0.049 | 0.052 | 0.025 | 52.664 | 0.011 | 0.872 |
| **22** | HR | beta | Vis | -0.042 | 0.302 | 0.041 | 200.527 | 0.006 | 0.467 |
| **23** | HR | beta | SM | -0.017 | 0.002 | 0.005 | -260.523 | 0.024 | 0.768 |
| **24** | HR | beta | DAN | 0.007 | 0.302 | 0.007 | -223.090 | 0.002 | 0.893 |
| **25** | HR | beta | VAN | -0.014 | 0.046 | 0.007 | -203.434 | 0.007 | 0.855 |
| **26** | HR | beta | Lim | -0.001 | 0.899 | 0.011 | -105.369 | 0.000 | 0.543 |
| **27** | HR | beta | FPN | 0.021 | 0.364 | 0.024 | 91.460 | 0.002 | 0.740 |
| **28** | HR | beta | DMN | -0.040 | 0.321 | 0.040 | 211.824 | 0.005 | 0.447 |
| **29** | HR | gamma | Vis | -0.009 | 0.160 | 0.006 | -224.964 | 0.006 | 0.689 |
| **30** | HR | gamma | SM | -0.002 | 0.729 | 0.006 | -210.153 | 0.000 | 0.854 |
| **31** | HR | gamma | DAN | -0.019 | 0.019 | 0.008 | -173.508 | 0.011 | 0.847 |
| **32** | HR | gamma | VAN | -0.010 | 0.309 | 0.010 | -113.416 | 0.004 | 0.564 |
| **33** | HR | gamma | Lim | 0.048 | 0.051 | 0.025 | 84.729 | 0.010 | 0.807 |
| **34** | HR | gamma | FPN | -0.015 | 0.727 | 0.042 | 211.119 | 0.001 | 0.435 |
| **35** | HR | gamma | DMN | -0.014 | 0.069 | 0.008 | -235.725 | 0.013 | 0.797 |
| **36** | FAR | delta | Vis | 0.010 | 0.128 | 0.006 | -214.062 | 0.004 | 0.865 |
| **37** | FAR | delta | SM | -0.016 | 0.020 | 0.007 | -194.115 | 0.008 | 0.875 |
| **38** | FAR | delta | DAN | -0.010 | 0.303 | 0.010 | -119.223 | 0.003 | 0.654 |
| **39** | FAR | delta | VAN | 0.043 | 0.066 | 0.024 | 76.287 | 0.008 | 0.833 |
| **40** | FAR | delta | Lim | -0.011 | 0.783 | 0.040 | 209.238 | 0.000 | 0.502 |
| **41** | FAR | delta | FPN | -0.006 | 0.386 | 0.007 | -233.704 | 0.003 | 0.771 |
| **42** | FAR | delta | DMN | 0.006 | 0.414 | 0.007 | -204.861 | 0.001 | 0.861 |
| **43** | FAR | theta | Vis | 0.000 | 0.984 | 0.006 | -200.456 | 0.000 | 0.868 |
| **44** | FAR | theta | SM | 0.004 | 0.634 | 0.008 | -145.608 | 0.001 | 0.691 |
| **45** | FAR | theta | DAN | -0.008 | 0.617 | 0.017 | 43.992 | 0.000 | 0.851 |
| **46** | FAR | theta | VAN | -0.021 | 0.575 | 0.037 | 192.952 | 0.001 | 0.472 |
| **47** | FAR | theta | Lim | 0.003 | 0.546 | 0.006 | -235.668 | 0.001 | 0.706 |
| **48** | FAR | theta | FPN | 0.006 | 0.372 | 0.006 | -194.822 | 0.001 | 0.826 |
| **49** | FAR | theta | DMN | 0.000 | 0.954 | 0.006 | -194.013 | 0.000 | 0.852 |
| **50** | FAR | alpha | Vis | 0.001 | 0.881 | 0.008 | -157.775 | 0.000 | 0.728 |
| **51** | FAR | alpha | SM | -0.008 | 0.715 | 0.022 | 67.833 | 0.000 | 0.823 |
| **52** | FAR | alpha | DAN | -0.016 | 0.228 | 0.013 | 114.419 | 0.002 | 0.566 |
| **53** | FAR | alpha | VAN | 0.001 | 0.829 | 0.007 | -208.452 | 0.000 | 0.616 |
| **54** | FAR | alpha | Lim | 0.000 | 0.975 | 0.006 | -207.589 | 0.000 | 0.854 |
| **55** | FAR | alpha | FPN | -0.005 | 0.479 | 0.007 | -190.132 | 0.001 | 0.837 |
| **56** | FAR | alpha | DMN | -0.015 | 0.126 | 0.010 | -125.469 | 0.010 | 0.578 |
| **57** | FAR | beta | Vis | 0.030 | 0.206 | 0.024 | 71.603 | 0.005 | 0.785 |
| **58** | FAR | beta | SM | 0.040 | 0.307 | 0.039 | 206.903 | 0.005 | 0.456 |
| **59** | FAR | beta | DAN | -0.007 | 0.300 | 0.006 | -238.126 | 0.004 | 0.727 |
| **60** | FAR | beta | VAN | -0.006 | 0.316 | 0.006 | -212.742 | 0.001 | 0.843 |
| **61** | FAR | beta | Lim | -0.019 | 0.041 | 0.009 | -173.615 | 0.010 | 0.862 |
| **62** | FAR | beta | FPN | -0.012 | 0.208 | 0.010 | -120.841 | 0.005 | 0.600 |
| **63** | FAR | beta | DMN | 0.052 | 0.038 | 0.025 | 86.962 | 0.011 | 0.806 |
| **64** | FAR | gamma | Vis | -0.008 | 0.853 | 0.042 | 197.998 | 0.000 | 0.501 |
| **65** | FAR | gamma | SM | -0.014 | 0.072 | 0.008 | -234.953 | 0.013 | 0.788 |
| **66** | FAR | gamma | DAN | 0.007 | 0.321 | 0.007 | -226.446 | 0.002 | 0.889 |
| **67** | FAR | gamma | VAN | -0.016 | 0.045 | 0.008 | -213.481 | 0.008 | 0.909 |
| **68** | FAR | gamma | Lim | -0.002 | 0.806 | 0.008 | -153.536 | 0.000 | 0.732 |
| **69** | FAR | gamma | FPN | 0.029 | 0.228 | 0.024 | 72.090 | 0.003 | 0.843 |
| **70** | FAR | gamma | DMN | -0.034 | 0.383 | 0.040 | 180.813 | 0.004 | 0.596 |
| **71** | c | delta | Vis | -0.008 | 0.338 | 0.008 | -235.772 | 0.004 | 0.791 |
| **72** | c | delta | SM | 0.005 | 0.542 | 0.008 | -193.168 | 0.001 | 0.844 |
| **73** | c | delta | DAN | -0.010 | 0.160 | 0.007 | -194.561 | 0.003 | 0.863 |
| **74** | c | delta | VAN | 0.007 | 0.353 | 0.008 | -153.756 | 0.002 | 0.725 |
| **75** | c | delta | Lim | -0.003 | 0.904 | 0.021 | 64.502 | 0.000 | 0.838 |
| **76** | c | delta | FPN | -0.060 | 0.065 | 0.033 | 171.281 | 0.013 | 0.556 |
| **77** | c | delta | DMN | -0.002 | 0.749 | 0.006 | -239.653 | 0.000 | 0.718 |
| **78** | c | theta | Vis | 0.007 | 0.353 | 0.007 | -214.051 | 0.002 | 0.880 |
| **79** | c | theta | SM | -0.005 | 0.411 | 0.006 | -188.076 | 0.001 | 0.839 |
| **80** | c | theta | DAN | 0.002 | 0.812 | 0.009 | -134.007 | 0.000 | 0.679 |
| **81** | c | theta | VAN | 0.000 | 0.983 | 0.022 | 79.150 | 0.000 | 0.804 |
| **82** | c | theta | Lim | -0.031 | 0.375 | 0.035 | 184.327 | 0.003 | 0.542 |
| **83** | c | theta | FPN | -0.004 | 0.534 | 0.007 | -246.263 | 0.001 | 0.750 |
| **84** | c | theta | DMN | 0.004 | 0.501 | 0.006 | -230.571 | 0.001 | 0.892 |
| **85** | c | alpha | Vis | -0.006 | 0.404 | 0.007 | -194.257 | 0.001 | 0.850 |
| **86** | c | alpha | SM | -0.014 | 0.162 | 0.010 | -114.150 | 0.008 | 0.537 |
| **87** | c | alpha | DAN | 0.029 | 0.203 | 0.023 | 79.464 | 0.004 | 0.769 |
| **88** | c | alpha | VAN | 0.025 | 0.521 | 0.039 | 205.364 | 0.002 | 0.460 |
| **89** | c | alpha | Lim | -0.006 | 0.273 | 0.006 | -250.170 | 0.003 | 0.755 |
| **90** | c | alpha | FPN | -0.001 | 0.795 | 0.005 | -248.555 | 0.000 | 0.891 |
| **91** | c | alpha | DMN | -0.021 | 0.021 | 0.009 | -194.810 | 0.014 | 0.884 |
| **92** | c | beta | Vis | -0.003 | 0.718 | 0.009 | -135.544 | 0.000 | 0.636 |
| **93** | c | beta | SM | 0.039 | 0.085 | 0.023 | 72.130 | 0.007 | 0.824 |
| **94** | c | beta | DAN | -0.045 | 0.282 | 0.042 | 193.616 | 0.007 | 0.482 |
| **95** | c | beta | VAN | -0.016 | 0.048 | 0.008 | -228.795 | 0.016 | 0.776 |
| **96** | c | beta | Lim | 0.008 | 0.150 | 0.006 | -219.028 | 0.003 | 0.858 |
| **97** | c | beta | FPN | -0.019 | 0.009 | 0.007 | -180.307 | 0.011 | 0.849 |
| **98** | c | beta | DMN | -0.003 | 0.740 | 0.009 | -149.679 | 0.000 | 0.729 |
| **99** | c | gamma | Vis | 0.037 | 0.092 | 0.022 | 63.191 | 0.006 | 0.849 |
| **100** | c | gamma | SM | -0.040 | 0.351 | 0.043 | 203.337 | 0.004 | 0.522 |
| **101** | c | gamma | DAN | -0.009 | 0.272 | 0.008 | -218.246 | 0.005 | 0.739 |
| **102** | c | gamma | VAN | 0.004 | 0.502 | 0.006 | -214.108 | 0.001 | 0.860 |
| **103** | c | gamma | Lim | 0.000 | 0.967 | 0.006 | -216.593 | 0.000 | 0.891 |
| **104** | c | gamma | FPN | 0.008 | 0.297 | 0.008 | -155.929 | 0.003 | 0.719 |
| **105** | c | gamma | DMN | -0.015 | 0.400 | 0.018 | 55.061 | 0.001 | 0.837 |
| **106** | d | delta | Vis | -0.040 | 0.256 | 0.035 | 183.586 | 0.005 | 0.511 |
| **107** | d | delta | SM | 0.002 | 0.716 | 0.006 | -222.515 | 0.000 | 0.675 |
| **108** | d | delta | DAN | 0.003 | 0.599 | 0.006 | -202.196 | 0.000 | 0.834 |
| **109** | d | delta | VAN | -0.004 | 0.529 | 0.006 | -220.401 | 0.000 | 0.896 |
| **110** | d | delta | Lim | 0.002 | 0.785 | 0.008 | -154.462 | 0.000 | 0.704 |
| **111** | d | delta | FPN | -0.008 | 0.685 | 0.019 | 50.899 | 0.000 | 0.845 |
| **112** | d | delta | DMN | -0.003 | 0.952 | 0.046 | 168.179 | 0.005 | 0.519 |
| **113** | d | theta | Vis | 0.000 | 0.981 | 0.006 | -239.891 | 0.000 | 0.731 |
| **114** | d | theta | SM | -0.001 | 0.892 | 0.006 | -216.232 | 0.000 | 0.861 |
| **115** | d | theta | DAN | -0.009 | 0.191 | 0.007 | -191.608 | 0.003 | 0.831 |
| **116** | d | theta | VAN | -0.013 | 0.207 | 0.010 | -133.544 | 0.007 | 0.609 |
| **117** | d | theta | Lim | 0.036 | 0.153 | 0.025 | 80.129 | 0.006 | 0.765 |
| **118** | d | theta | FPN | 0.029 | 0.440 | 0.037 | 195.706 | 0.003 | 0.475 |
| **119** | d | theta | DMN | -0.010 | 0.098 | 0.006 | -235.985 | 0.008 | 0.698 |
| **120** | d | alpha | Vis | -0.003 | 0.636 | 0.006 | -222.102 | 0.000 | 0.857 |
| **121** | d | alpha | SM | -0.011 | 0.154 | 0.008 | -180.704 | 0.004 | 0.854 |
| **122** | d | alpha | DAN | 0.003 | 0.755 | 0.009 | -123.802 | 0.000 | 0.612 |
| **123** | d | alpha | VAN | 0.008 | 0.716 | 0.021 | 87.408 | 0.000 | 0.788 |
| **124** | d | alpha | Lim | -0.048 | 0.189 | 0.037 | 191.060 | 0.008 | 0.481 |
| **125** | d | alpha | FPN | -0.018 | 0.007 | 0.007 | -256.416 | 0.024 | 0.798 |
| **126** | d | alpha | DMN | 0.004 | 0.489 | 0.005 | -227.574 | 0.001 | 0.857 |
| **127** | d | beta | Vis | -0.014 | 0.054 | 0.007 | -214.458 | 0.006 | 0.904 |
| **128** | d | beta | SM | -0.007 | 0.494 | 0.010 | -134.011 | 0.002 | 0.583 |
| **129** | d | beta | DAN | 0.022 | 0.277 | 0.020 | 72.651 | 0.002 | 0.796 |
| **130** | d | beta | VAN | -0.033 | 0.352 | 0.036 | 185.284 | 0.004 | 0.474 |
| **131** | d | beta | Lim | -0.013 | 0.054 | 0.007 | -252.395 | 0.014 | 0.790 |
| **132** | d | beta | FPN | 0.008 | 0.166 | 0.006 | -220.185 | 0.002 | 0.856 |
| **133** | d | beta | DMN | -0.003 | 0.697 | 0.006 | -222.651 | 0.000 | 0.905 |
| **134** | d | gamma | Vis | 0.005 | 0.590 | 0.009 | -140.320 | 0.001 | 0.665 |
| **135** | d | gamma | SM | -0.008 | 0.681 | 0.019 | 63.476 | 0.000 | 0.825 |
| **136** | d | gamma | DAN | -0.033 | 0.323 | 0.033 | 172.357 | 0.004 | 0.520 |
| **137** | d | gamma | VAN | -0.001 | 0.795 | 0.006 | -260.619 | 0.000 | 0.767 |
| **138** | d | gamma | Lim | 0.003 | 0.660 | 0.006 | -198.828 | 0.000 | 0.830 |
| **139** | d | gamma | FPN | -0.008 | 0.300 | 0.008 | -214.713 | 0.002 | 0.898 |
| **140** | d | gamma | DMN | 0.007 | 0.437 | 0.009 | -147.815 | 0.002 | 0.654 |
| **141** | accuracy | delta | Vis | -0.012 | 0.544 | 0.019 | 62.501 | 0.001 | 0.808 |
| **142** | accuracy | delta | SM | -0.065 | 0.049 | 0.033 | 169.846 | 0.016 | 0.532 |
| **143** | accuracy | delta | DAN | -0.003 | 0.562 | 0.005 | -245.924 | 0.001 | 0.709 |
| **144** | accuracy | delta | VAN | 0.003 | 0.650 | 0.007 | -216.769 | 0.000 | 0.877 |
| **145** | accuracy | delta | Lim | -0.014 | 0.043 | 0.007 | -210.567 | 0.007 | 0.856 |
| **146** | accuracy | delta | FPN | 0.000 | 0.982 | 0.008 | -148.598 | 0.000 | 0.677 |
| **147** | accuracy | delta | DMN | 0.008 | 0.657 | 0.019 | 51.229 | 0.000 | 0.825 |
| **148** | accuracy | theta | Vis | -0.042 | 0.243 | 0.036 | 184.814 | 0.006 | 0.475 |
| **149** | accuracy | theta | SM | -0.016 | 0.002 | 0.005 | -266.238 | 0.023 | 0.748 |
| **150** | accuracy | theta | DAN | 0.005 | 0.487 | 0.007 | -192.193 | 0.001 | 0.816 |
| **151** | accuracy | theta | VAN | -0.020 | 0.029 | 0.009 | -198.222 | 0.013 | 0.898 |
| **152** | accuracy | theta | Lim | -0.006 | 0.549 | 0.010 | -134.263 | 0.001 | 0.661 |
| **153** | accuracy | theta | FPN | 0.043 | 0.054 | 0.022 | 73.113 | 0.008 | 0.828 |
| **154** | accuracy | theta | DMN | -0.037 | 0.408 | 0.044 | 189.769 | 0.004 | 0.573 |
| **155** | accuracy | alpha | Vis | -0.018 | 0.016 | 0.008 | -258.485 | 0.022 | 0.839 |
| **156** | accuracy | alpha | SM | 0.007 | 0.273 | 0.007 | -205.694 | 0.002 | 0.847 |
| **157** | accuracy | alpha | DAN | -0.017 | 0.046 | 0.008 | -182.420 | 0.008 | 0.870 |
| **158** | accuracy | alpha | VAN | -0.007 | 0.383 | 0.008 | -157.366 | 0.002 | 0.720 |
| **159** | accuracy | alpha | Lim | 0.042 | 0.053 | 0.022 | 47.892 | 0.008 | 0.872 |
| **160** | accuracy | alpha | FPN | -0.022 | 0.617 | 0.044 | 190.564 | 0.002 | 0.570 |
| **161** | accuracy | alpha | DMN | -0.010 | 0.194 | 0.008 | -229.409 | 0.007 | 0.766 |
| **162** | accuracy | beta | Vis | 0.005 | 0.417 | 0.007 | -221.123 | 0.001 | 0.872 |
| **163** | accuracy | beta | SM | -0.005 | 0.500 | 0.007 | -210.437 | 0.001 | 0.893 |
| **164** | accuracy | beta | DAN | 0.004 | 0.653 | 0.009 | -137.357 | 0.001 | 0.682 |
| **165** | accuracy | beta | VAN | 0.000 | 0.996 | 0.021 | 49.702 | 0.000 | 0.867 |
| **166** | accuracy | beta | Lim | -0.035 | 0.340 | 0.037 | 190.646 | 0.004 | 0.480 |
| **167** | accuracy | beta | FPN | -0.001 | 0.839 | 0.006 | -257.375 | 0.000 | 0.780 |
| **168** | accuracy | beta | DMN | 0.004 | 0.528 | 0.006 | -205.313 | 0.001 | 0.846 |
| **169** | accuracy | gamma | Vis | -0.009 | 0.172 | 0.007 | -200.376 | 0.003 | 0.870 |
| **170** | accuracy | gamma | SM | 0.001 | 0.901 | 0.009 | -125.528 | 0.000 | 0.646 |
| **171** | accuracy | gamma | DAN | 0.005 | 0.794 | 0.021 | 83.500 | 0.000 | 0.785 |
| **172** | accuracy | gamma | VAN | -0.046 | 0.197 | 0.036 | 189.582 | 0.007 | 0.510 |
| **173** | accuracy | gamma | Lim | -0.006 | 0.351 | 0.006 | -243.925 | 0.003 | 0.738 |
| **174** | accuracy | gamma | FPN | 0.002 | 0.742 | 0.006 | -245.053 | 0.000 | 0.906 |
| **175** | accuracy | gamma | DMN | -0.014 | 0.042 | 0.007 | -193.744 | 0.006 | 0.838 |
| **176** | RT | delta | Vis | -0.016 | 0.111 | 0.010 | -118.744 | 0.010 | 0.575 |
| **177** | RT | delta | SM | 0.048 | 0.052 | 0.025 | 74.951 | 0.012 | 0.794 |
| **178** | RT | delta | DAN | 0.025 | 0.527 | 0.039 | 205.520 | 0.002 | 0.450 |
| **179** | RT | delta | VAN | -0.014 | 0.023 | 0.006 | -224.367 | 0.015 | 0.680 |
| **180** | RT | delta | Lim | 0.007 | 0.255 | 0.006 | -209.911 | 0.002 | 0.843 |
| **181** | RT | delta | FPN | -0.020 | 0.035 | 0.009 | -182.145 | 0.013 | 0.870 |
| **182** | RT | delta | DMN | -0.005 | 0.545 | 0.009 | -136.102 | 0.001 | 0.631 |
| **183** | RT | theta | Vis | 0.038 | 0.102 | 0.024 | 74.986 | 0.007 | 0.814 |
| **184** | RT | theta | SM | -0.041 | 0.273 | 0.038 | 196.530 | 0.006 | 0.462 |
| **185** | RT | theta | DAN | -0.018 | 0.020 | 0.008 | -224.745 | 0.021 | 0.758 |
| **186** | RT | theta | VAN | 0.008 | 0.169 | 0.006 | -238.468 | 0.002 | 0.890 |
| **187** | RT | theta | Lim | -0.020 | 0.019 | 0.008 | -201.825 | 0.012 | 0.893 |
| **188** | RT | theta | FPN | -0.005 | 0.526 | 0.008 | -149.081 | 0.001 | 0.699 |
| **189** | RT | theta | DMN | 0.039 | 0.090 | 0.023 | 55.872 | 0.007 | 0.858 |
| **190** | RT | alpha | Vis | -0.043 | 0.294 | 0.041 | 182.177 | 0.006 | 0.585 |
| **191** | RT | alpha | SM | -0.013 | 0.071 | 0.007 | -240.555 | 0.011 | 0.785 |
| **192** | RT | alpha | DAN | 0.011 | 0.077 | 0.006 | -207.157 | 0.004 | 0.845 |
| **193** | RT | alpha | VAN | -0.011 | 0.127 | 0.007 | -203.758 | 0.004 | 0.887 |
| **194** | RT | alpha | Lim | 0.014 | 0.122 | 0.009 | -134.241 | 0.006 | 0.679 |
| **195** | RT | alpha | FPN | -0.009 | 0.654 | 0.020 | 68.164 | 0.000 | 0.831 |
| **196** | RT | alpha | DMN | -0.085 | 0.024 | 0.038 | 192.689 | 0.023 | 0.499 |
| **197** | RT | beta | Vis | -0.007 | 0.240 | 0.006 | -255.383 | 0.004 | 0.784 |
| **198** | RT | beta | SM | 0.006 | 0.398 | 0.007 | -204.108 | 0.001 | 0.852 |
| **199** | RT | beta | DAN | -0.014 | 0.054 | 0.007 | -189.233 | 0.006 | 0.853 |
| **200** | RT | beta | VAN | 0.000 | 0.971 | 0.008 | -149.455 | 0.000 | 0.716 |
| **201** | RT | beta | Lim | 0.018 | 0.323 | 0.018 | 54.100 | 0.001 | 0.841 |
| **202** | RT | beta | FPN | -0.045 | 0.226 | 0.037 | 173.778 | 0.007 | 0.557 |
| **203** | RT | beta | DMN | -0.008 | 0.201 | 0.006 | -234.094 | 0.005 | 0.715 |
| **204** | RT | gamma | Vis | 0.004 | 0.488 | 0.006 | -246.248 | 0.001 | 0.905 |
| **205** | RT | gamma | SM | -0.014 | 0.041 | 0.007 | -191.275 | 0.007 | 0.833 |
| **206** | RT | gamma | DAN | -0.009 | 0.327 | 0.009 | -136.403 | 0.003 | 0.657 |
| **207** | RT | gamma | VAN | 0.032 | 0.151 | 0.022 | 73.766 | 0.005 | 0.799 |
| **208** | RT | gamma | Lim | -0.003 | 0.972 | 0.073 | 212.505 | 0.000 | 0.526 |
| **209** | RT | gamma | FPN | -0.018 | 0.005 | 0.006 | -236.743 | 0.025 | 0.738 |
| **210** | RT | gamma | DMN | 0.002 | 0.788 | 0.006 | -215.792 | 0.000 | 0.863 |
| Supplementary table 2. Statistics for Figure 5B. | | | | | | | | | |
